# Adenovirus-prime and baculovirus-boost heterologous immunization achieves sterile protection against malaria sporozoite challenge in a murine model

**DOI:** 10.1038/s41598-018-21369-y

**Published:** 2018-03-01

**Authors:** Kunitaka Yoshida, Mitsuhiro Iyori, Andrew M. Blagborough, Ahmed M. Salman, Pawan Dulal, Katarzyna A. Sala, Daisuke S. Yamamoto, Shahid M. Khan, Chris J. Janse, Sumi Biswas, Tatsuya Yoshii, Yenni Yusuf, Masaharu Tokoro, Adrian V. S. Hill, Shigeto Yoshida

**Affiliations:** 1 Laboratory of Vaccinology and Applied Immunology, Kanazawa University School of Pharmacy, Kakuma-machi, Kanazawa 920-1192 Japan; 2 Kanazawa University Graduate School of Medical Sciences, 13 Takara-machi, Kanazawa, 920-0934 Japan; 3 Department of Life Sciences, Sir Alexander Fleming Building, Imperial College London, Imperial College Road, South Kensington, London SW7 2AZ UK; 4 The Jenner Institute, Nuffield Department of Medicine, University of Oxford, Old Road Campus Research Building, Roosevelt Drive, Oxford, OX3 7DQ UK; 5 Leiden Malaria Research Group, Department of Parasitology, Center of Infectious Diseases, Leiden University Medical Center, (LUMC, L4-Q), Albinusdreef 2, 2333 ZA Leiden, The Netherlands; 6 Division of Medical Zoology, Department of Infection and Immunity, Jichi Medical University, 3311-1 Yakushiji, Shimotsuke, 329-0431 Tochigi Japan

## Abstract

With the increasing prevalence of artemisinin-resistant malaria parasites, a highly efficacious and durable vaccine for malaria is urgently required. We have developed an experimental virus-vectored vaccine platform based on an envelope-modified baculovirus dual-expression system (emBDES). Here, we show a conceptually new vaccine platform based on an adenovirus-prime/emBDES-boost heterologous immunization regimen expressing the *Plasmodium falciparum* circumsporozoite protein (PfCSP). A human adenovirus 5-prime/emBDES-boost heterologous immunization regimen consistently achieved higher sterile protection against transgenic *P. berghei* sporozoites expressing PfCSP after a mosquito-bite challenge than reverse-ordered or homologous immunization. This high protective efficacy was also achieved with a chimpanzee adenovirus 63-prime/emBDES-boost heterologous immunization regimen against an intravenous sporozoite challenge. Thus, we show that the adenovirus-prime/emBDES-boost heterologous immunization regimen confers sterile protection against sporozoite challenge by two individual routes, providing a promising new malaria vaccine platform for future clinical use.

## Introduction

Malaria remains a severe public health problem and causes significant economic losses worldwide. Nearly half the world’s population is at risk of malaria, with a disproportionate risk and high mortality in children under 5 years old. In 2016, there were approximately 216 million cases of malaria and an estimated 445,000 malaria deaths^1^. The World Health Organization recommends the use of artemisinin as the core compound of a combination treatment, but artemisinin resistance is already present in some countries in South-East Asia^1^. A malaria vaccine is an attractive alternative to drug treatment or prophylaxis. The most advanced candidate *Plasmodium falciparum* malaria vaccine, RTS,S/AS01 (also known as Mosquirix™), based on the *P. falciparum* circumsporozoite protein (PfCSP) targeting the pre-erythrocytic stage, conferred limited protection (18–26% in infants) in a phase III trial in sub-Saharan Africa^2,3^. Although the mechanism of the RTS,S/AS01-induced protective immune response has not been clarified in detail, the CSP-specific antibodies (Abs) and CD4^+^ T-cell responses induced by vaccination with RTS,S/AS01 have been correlated with protection^4,5^. To improve the protective efficacy of RTS,S/AS01, an adenovirus 35 prime and RTS,S/AS01 boost heterologous immunization regimen followed by another booster dose of RTS,S/AS01 (ARR) was tested in humans^6^. Although ARR immunization enhanced the CD4^+^ and CD8^+^ T-cell responses better than three doses of RTS,S/AS01 (RRR), the protective efficacy of ARR immunization did not exceed that of RRR immunization^6,7^. Future strategies that surpass RTS,S/AS01-induced protection may require alternative highly immunogenic prime-boost regimens and/or additional target antigens. Therefore, the development of viral vectors as vaccine platforms continues to be important.

We have developed a new viral-vectored vaccine system based on the baculovirus *Autographa californica* nucleopolyhedrosis virus, called the ‘baculovirus dual-expression system’ (BDES). BDES drives the expression of malaria antigen with a dual promoter that consists of both baculovirus-derived polyhedrin and mammal-derived cytomegalovirus promoters, which allow the antigen to be displayed in its native conformation on the viral envelope and to be expressed after the transduction of mammalian cells, respectively^8^. Therefore, BDES functions as both a component vaccine and a DNA vaccine. We have shown that BDES is an effective malaria vaccine platform for all three stages of the *Plasmodium* life cycle, including the pre-erythrocytic stage^8–10^, asexual blood stage^11,12^, and sexual stage^9,13–15^, when transgenic *P. berghei* parasites expressing human *Plasmodium* antigens were used for its evaluation. In addition to the high efficacy of BDES demonstrated in these experiments, BDES-based PfCSP vaccines (BDES-PfCSP) have been shown to be safe and well tolerated in Rhesus monkeys, with acceptable reactogenicity and systemic toxicity^10^. More recently, we have generated an envelope-modified BDES (emBDES-PfCSP) pre-erythrocytic-stage vaccine, which displays both human decay-accelerating factor (DAF) and *P. falciparum* circumsporozoite protein (PfCSP) on the virion surface^16^. The DAF-shielded emBDES induced enhanced resistance to serum inactivation, and when combined with an interleukin 12 (IL-12)-expressing baculovirus vaccine (emBDES-PfCSP/IL12), further enhanced the protective efficacy against sporozoite challenge in a murine model after two or three boosts^17^. However, to ensure its subsequent field application and improve its cost-effectiveness, a simpler immunization regimen (e.g., vaccine dose) and improved protective efficacy in terms of the T-cell-mediated immune responses are required.

Several studies have shown the efficacy of heterologous prime-boost immunization strategies in inducing T-cell-mediated immunity against a variety of pathogens, including *Mycobacterium tuberculosis*^18–23^, human immunodeficiency virus^24,25^, and Ebola virus^26,27^. Researchers in malaria at the Jenner Institute (University of Oxford, Oxford, UK) have demonstrated that a heterologous prime-boost immunization regimen using a replication-deficient chimpanzee adenovirus 63 (ChAd63) and modified vaccinia virus Ankara (MVA) increased the number of antigen-specific CD8^+^ T cells, improving the protective efficacy in mouse and macaque models^28–30^. It is widely recognized that a successful pre-erythrocytic malaria vaccine must induce not only humoral immune responses but also CD8^+^ T-cell-mediated immune responses^31–35^ to inhibit the infection of the liver by sporozoites and to eliminate any resulting liver-stage parasites that develop in hepatocytes.

In this study, we combined the baculoviral-vectored vaccine, emBDES-PfCSP/IL12, with a human adenovirus 5 (AdHu5)-based PfCSP vaccine (AdHu5-PfCSP) or ChAd63-based PfCSP vaccine (ChAd63-PfCSP) to determine whether the protective efficacy was improved, and to identify immunological surrogates for protection. We demonstrate that the new vaccine platform using an adenovirus-prime and emBDES-boost regimen elicited higher protective efficacy against sporozoite challenge than the reverse-ordered or homologous immunization. This immunization regimen also functions as a ‘hybrid’ vaccine platform that effectively induces both humoral and cellular immune responses. These findings illustrate a novel hybrid malaria vaccine based on an adenovirus vaccine and an emBDES vaccine, which has unique anti-parasite advantages and potential utility as a ‘next-generation’ clinical candidate vaccine against other emerging and re-emerging infectious diseases.

## Results

### Reduction of transgene expression following re-administration of a baculoviral vector

**Figure 1:**
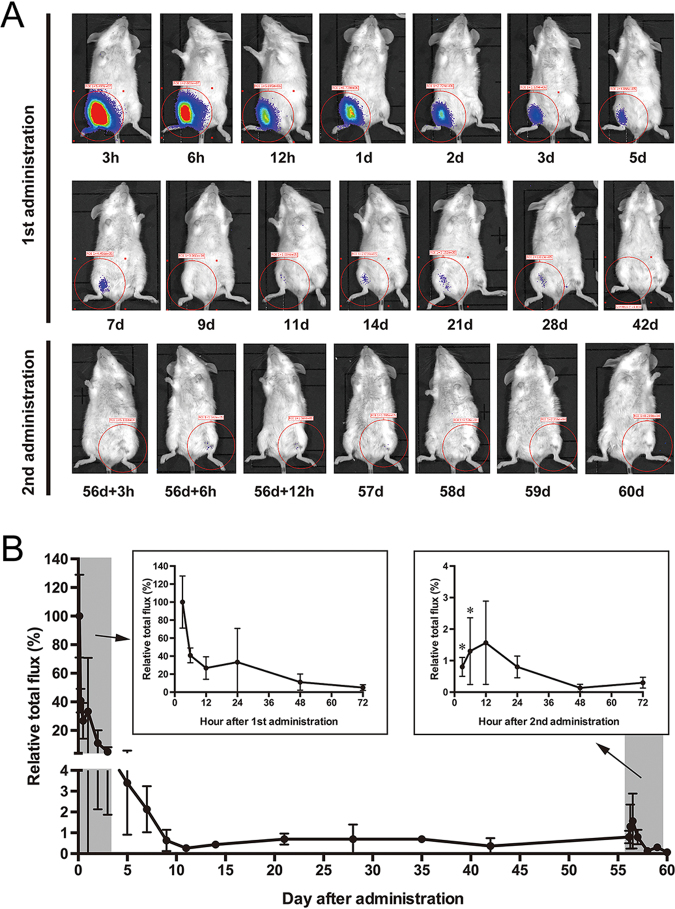
Reduction in the transduction efficacy of baculovirus *in vivo* after re-administration. (**A**) Luciferase expression at different time points, detected by using the IVIS Lumina LT Series III *In Vivo* imaging system. Luciferase-expressing BES-GL3 was administered into the right tibialis anterior muscle of BALB/c mice (n = 3; 1 × 10^8^ pfu/mouse) on day 0. Luciferase expression was reduced to an undetectable level on day 42, and BES-GL3 was re-administered into the left tibialis anterior muscle on day 56 (14 days later). (**B**) Quantitative analysis of luciferase expression. Total flux at different time points was normalized to that at 3 h and is expressed as relative total flux. Asterisks indicate a significant difference (*p* < 0.05) on Student’s t-test compared with first administration at that time point.

We have previously shown that emBDES-PfCSP/IL12 requires 3–4 doses of immunization to elicit 60–80% sterile protection against sporozoite challenge^17^. To examine the transduction efficacy of baculovirus *in vivo* after its re-administration, BES-GL3 expressing the luciferase gene was administered into the right tibialis anterior muscle of BALB/c mice (1 × 10^8^ PFU/mouse; n = 3) on day 0. Luciferase expression was monitored with bioluminescence imaging (Fig. 1A), and the data for the total flux (Fig. 1B) at different time points were normalized against the total flux after 3 h (defined as 100%). The expression of luciferase was initially robust but rapidly decreased to 2% on day 7 and had disappeared on day 42. When BES-GL3 was re-administered into the left tibialis anterior muscle on day 56, its expression (56d + 3 h) was severely impaired, decreasing to almost 1% of that at 3 h after the first administration into the right tibialis anterior muscle (*p* < 0.05, Student’s t-test) (Figs. 1A and B). Similar to the results for other viral vectors, these results indicate that multiple administrations of baculovirus dampen the vaccine-antigen-specific booster response because the vector itself is rapidly cleared by the immune system induced by the prime immunization.

### High levels of PfCSP expression by AdHu5-PfCSP on the surface of infected cells

To overcome the potential problem caused by multiple administrations of baculovirus, we investigated a new prime-boost immunization regimen using emBDES in combination with other vaccine vectors. We first chose replication-deficient AdHu5 as the partner of emBDES because the recombinant AdHu5 is one of the most efficient vectors in inducing T-cell responses, which may play an important role in the elimination of pre-erythrocytic parasites.

**Figure 2:**
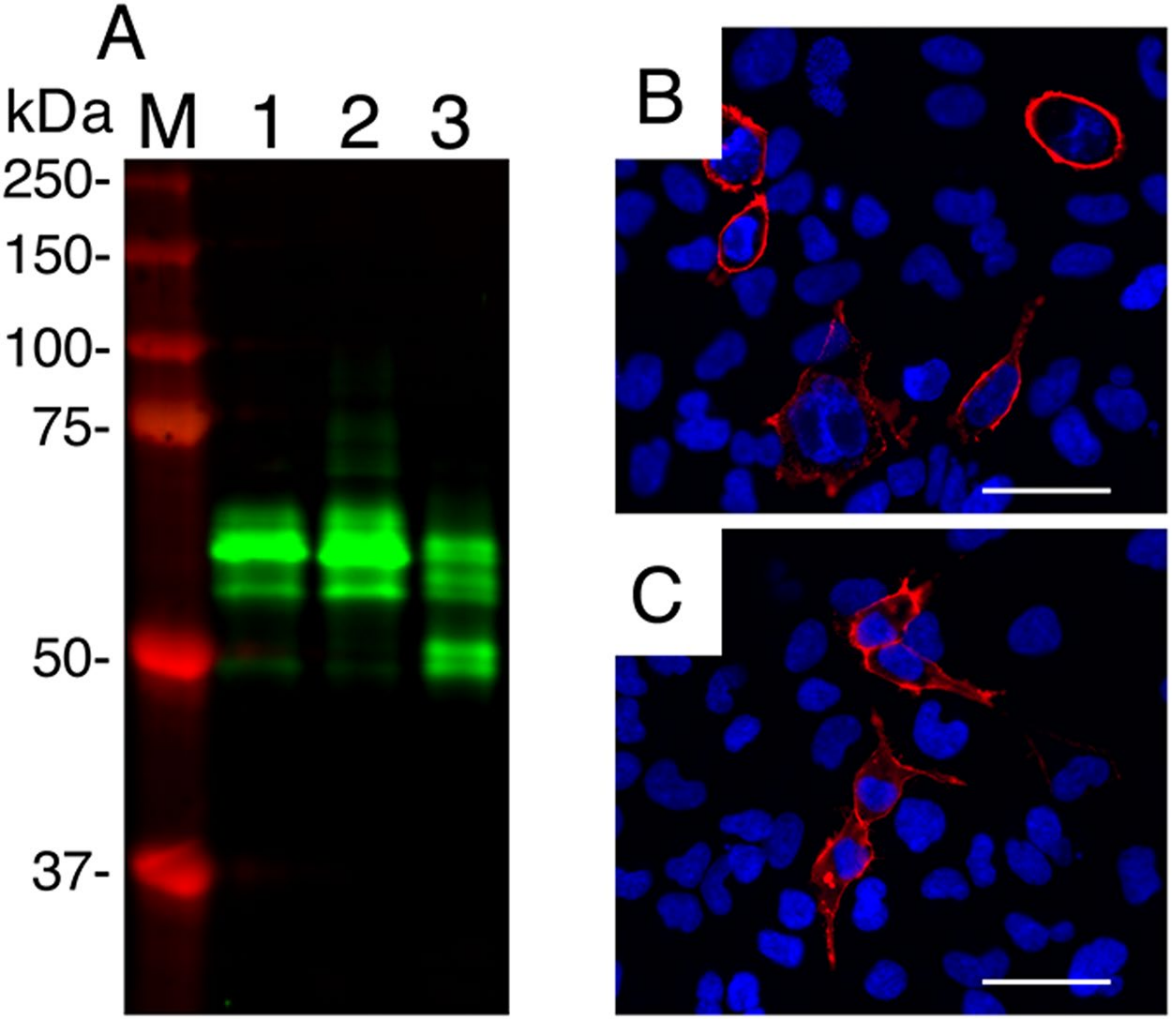
Expression of PfCSP in mammalian cells. (**A**) Immunoblotting analysis of HEK293A cells infected with AdHu5-PfCSP (MOI = 1) (lane 1), or transduced with emBDES-PfCSP (MOI = 100) (lane 2) or emBDES-PfCSP viral particles (1 × 10^6^ pfu) (lane 3). Cells or viral particles were lysed, loaded onto a 10% SDS-PAGE and immunoblotted with anti-PfCSP mAb 2A10. M, molecular marker. (**B**) HEK293A cells (3 × 10^4^) were infected with AdHu5-PfCSP (MOI = 1). (**C**) HEK293A cells were transduced with emBDES-PfCSP (MOI = 100). After 48 h, the cells were fixed with 4% paraformaldehyde, and incubated with Alexa-Fluor-594-conjugated anti-PfCSP mAb 2A10. Cell nuclei were visualized with 4′,6-diamidino-2-phenylindole (DAPI; blue). Original magnification, ×400. Bars = 50 μm.

We constructed an AdHu5-PfCSP vaccine carrying the same gene cassette as emBDES-PfCSP, which encodes a glycosylphosphatidylinositol (GPI)-anchor lacking PfCSP (Leu19-Val377) fused to the VSV-G protein membrane anchor sequence, followed by a *wpre* sequence, under the control of the CAG promoter. Immunoblotting showed that AdHu5-PfCSP [multiplicity of infection (MOI) = 1] expressed similar levels of the PfCSP-VSV-G fusion protein, with the predicted *Mr* of 53 kDa, as emBDES-PfCSP (MOI = 100) (Fig. 2A, lanes 1 and 2) and emBDES-PfCSP incorporated PfCSP into the viral virion (Fig. 2A, lane 3). In an indirect immunofluorescent antibody (IFA) staining, non-permeabilized cells infected with AdHu5-PfCSP (MOI = 1) or transduced with emBDES-PfCSP (MOI = 100) were incubated with an anti-PfCSP monoclonal antibody (mAb) 2A10 conjugated with Alexa Fluor 594. As expected, strong immunofluorescent signals were detected on the surfaces of both the AdHu5-PfCSP-infected and emBDES-PfCSP-transduced cells, indicating that VSV-G was anchored to the cell membranes (Fig. 2B,C).

### Immunogenicity of heterologous prime-boost regimens using AdHu5-PfCSP and emBDES-PfCSP/IL12

**Figure 3:**
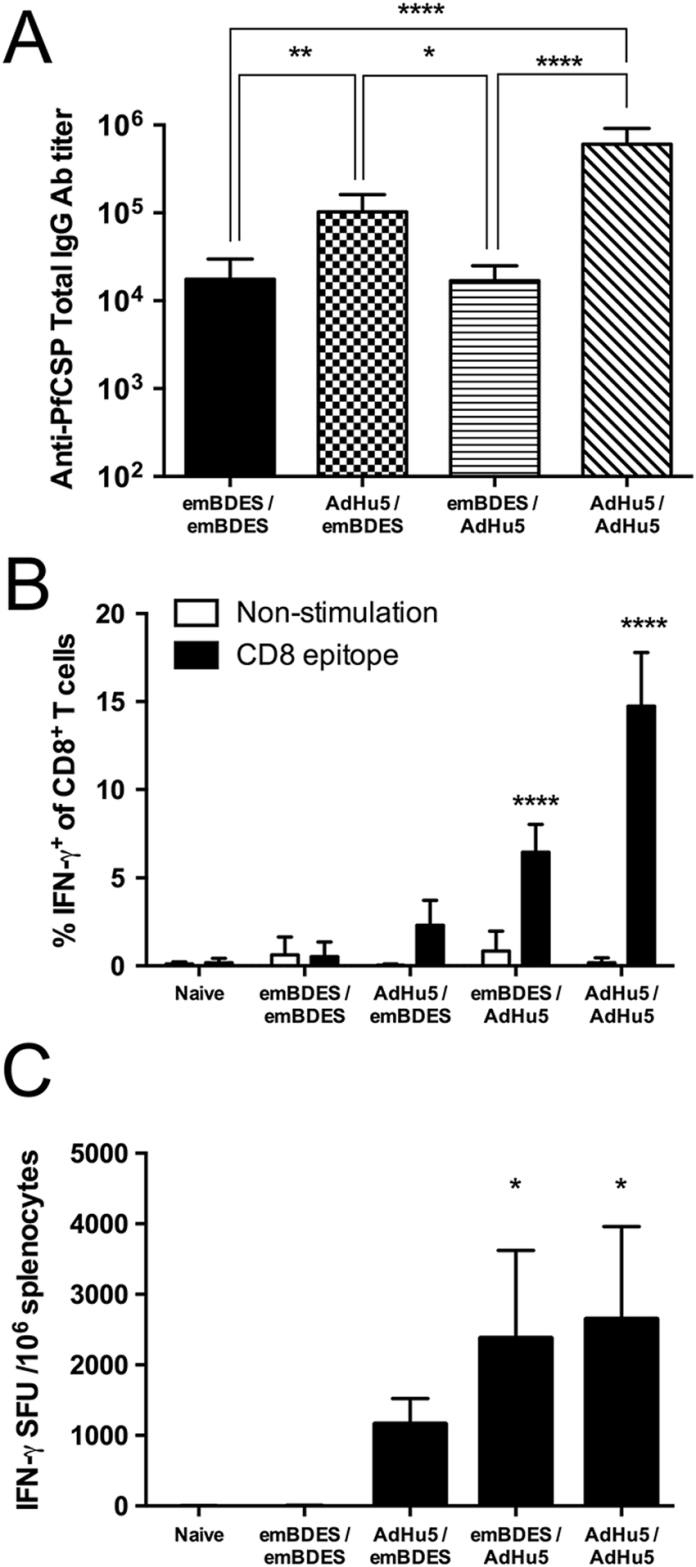
PfCSP-specific Abs and cellular immune responses in mice immunized with heterologous prime-boost regimens using AdHu5-PfCSP and emBDES-PfCSP/IL12. (**A**) Anti-PfCSP IgG Ab responses. BALB/c mice were immunized with the indicated regimens. AdHu5-PfCSP and emBDES-PfCSP/IL12 are shown as AdHu5 and emBDES, respectively. Two weeks after boosting, individual sera were collected and their anti-PfCSP IgG Ab titres were determined with ELISA (Data from experiment 1 are shown in Table 1). Bars and error bars indicate the means and SD of the values, respectively. Between-group differences were assessed with the Kruskal-Wallis test with Dunn’s correction for multiple comparisons. ****p < 0.0001, **p < 0.01, and *p < 0.05. (**B**,**C**) PfCSP-specific cellular immune responses. Two weeks after boosting with the indicated regimens, splenocytes were stimulated with the synthetic PfCSP-specific CD8 T-cell epitope. (**B**) An ICS assay was performed on the splenocytes. Percentages of IFN-γ-secreting cells in the CD8^+^CD4^−^ T-cell population are shown after the subtraction of the percentages of cells stained with an isotype control Ab. Bars and error bars indicate means and SD, respectively; n = 3 or 4. Between-group differences were assessed with the Kruskal-Wallis test with Dunn’s correction for multiple comparisons. ****p < 0.001, compared with naïve splenocytes stimulated with the same epitope. (**C**) An *ex vivo* ELISPOT assay was performed on splenocytes from the same mice. The IFN-γ SFU that reacted with the PfCSP-specific CD8 T-cell epitope per million splenocytes are shown. Bars and error bars indicate the mean numbers and SD, respectively. Between-group differences were assessed with the Kruskal-Wallis test with Dunn’s correction for multiple comparisons. *p < 0.05, compared with naïve splenocytes; n = 3 or 4.

The humoral and cellular immune responses induced by a heterologous regimen with AdHu5-PfCSP-prime/emBDES-PfCSP/IL12-boost were compared with those induced by a homologous regimen with either AdHu5-PfCSP or emBDES-PfCSP/IL12. Mice were immunized with various heterologous or homologous regimens using AdHu5-PfCSP and emBDES-PfCSP/IL12 at 3-week intervals. Two weeks after boosting, their sera were collected and the anti-PfCSP IgG antibody (Ab) titres were measured with an enzyme-linked immunosorbent assay (ELISA). The highest anti-PfCSP immunoglobulin G (IgG) Ab titre was induced when the homologous regimen with AdHu5-PfCSP was administered (Fig. 3A). The AdHu5-PfCSP-prime/emBDES-PfCSP/IL12-boost regimen induced significantly higher anti-PfCSP IgG Ab titres than did the emBDES-PfCSP/IL12-prime/AdHu5-PfCSP-boost regimen (Fig. 3A).

Similar to the humoral responses, the cellular immune responses, examined with intracellular cytokine staining (ICS) assays, showed that homologous regimen with AdHu5-PfCSP induced the highest interferon γ (IFN-γ) production by CD8^+^ T cells after stimulation with the CSP-derived *H-2K*^*d*^ peptide (Fig. 3B and Supplementary Fig. S1). The heterologous regimen with AdHu5-PfCSP and emBDES-PfCSP/IL12 induced significantly higher IFN-γ production than the homologous regimen with emBDES-PfCSP/IL12 (Fig. 3B). *Ex vivo* IFN-γ enzyme-linked immunosorbent spot (ELISPOT) assays also showed similar frequency patterns of IFN-γ-secreting cells [means of spot forming units (SFU) per million splenocytes: AdHu5-PfCSP, 2,648 > emBDES-PfCSP/IL12-prime/AdHu5-PfCSP-boost, 2,378 > AdHu5-PfCSP-prime/emBDES-PfCSP/IL12-boost, 1,162 > emBDES-PfCSP/IL12, 3.3] (Fig. 3C). These results indicate that emBDES-PfCSP/IL12 immunization with AdHu5-PfCSP as either the primary or booster immunization induces markedly stronger PfCSP-specific Ab and CD8^+^ T-cell responses than the homologous regimen with emBDES-PfCSP/IL12.

### Protective efficacy of heterologous prime-boost regimens using AdHu5-PfCSP and emBDES-PfCSP/IL12 against mosquito-bite challenge

**Figure 4:**
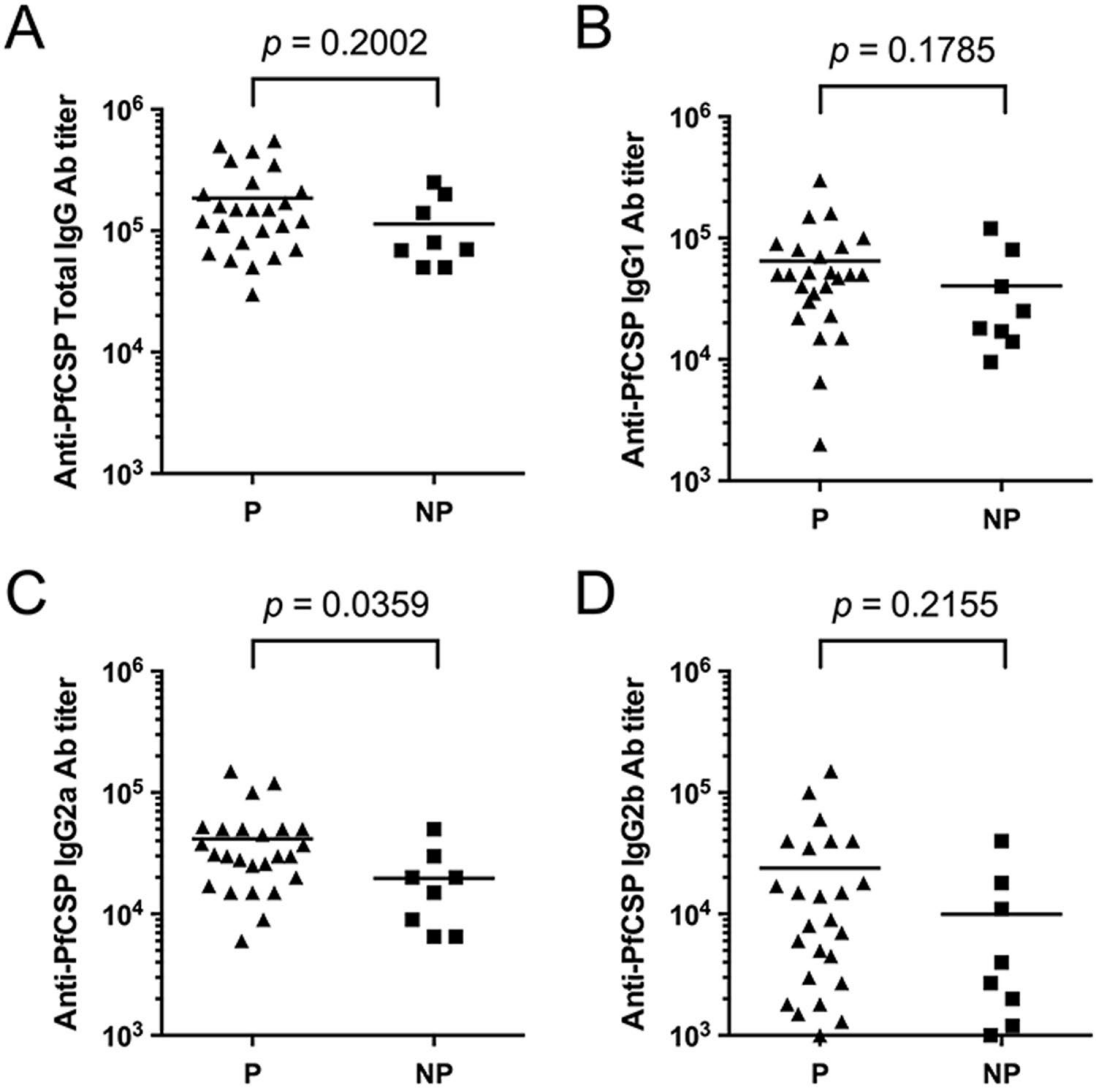
Comparison of anti-PfCSP IgG isotype Ab titres in mice immunized with AdHu5-PfCSP-prime/emBDES-PfCSP/IL12-boost regimen. All anti-PfCSP IgG isotype Ab titre data from mice immunized with AdHu5-prime/emBDES-boost regimen shown in Table 1 were combined and plotted separately for the protected (P) mice (n = 26) and unprotected (NP) mice (n = 8). Anti-PfCSP total IgG (**A**), IgG1 (**B**), IgG2a (**C**), and IgG2b Ab titres (**D**) are shown. Bars indicate mean values. The *p* values were determined with the Mann-Whitney test.

The protective efficacy of various heterologous and homologous prime-boost regimens using AdHu5-PfCSP and emBDES-PfCSP/IL12 was assessed by challenging immunized mice with the natural bites of mosquitoes infected with transgenic PfCSP-Tc/Pb sporozoites expressing full-length PfCSP. Although in this biting challenge the challenge dose presented to the mice is highly variable, there were no significant differences in the number of infectious bites among the groups (Supplementary Table S1). In each challenge, the AdHu5-PfCSP-prime/emBDES-PfCSP/IL12-boost regimen consistently conferred high sterile protection (50–100%), with a mean protective efficacy of 77% (Table 1). The reverse-ordered vaccination (emBDES-PfCSP/IL12-prime/AdHu5-PfCSP-boost) and homologous AdHu5-PfCSP regimens both displayed lower protective efficacy (each 20%; Table 1, Experiment 1). Among all the immunization groups, there was no significant correlation between the protection level and the PfCSP-specific immune responses (anti-PfCSP Ab titres and IFN-γ production). To identify surrogate marker(s) for protective efficacy, sera obtained from the group of mice immunized with the heterologous AdHu5-PfCSP-prime/emBDES-PfCSP/IL12-boost regimen before challenge were divided into the protected and unprotected groups, and their isotype IgGs were analysed (Fig. 4). Figure 4C shows that the protected mice had significantly higher anti-PfCSP IgG2a Ab titres than the unprotected mice (*p* = 0.0359). No correlation was observed between protection and the total IgG, IgG1 nor IgG2b Ab levels (Figs. 4A, B and D).

**Table 1:** The protective efficacy of heterologous prime-boost regimens using AdHu5 and emBDES against challenge by the bites of mosquitoes infected with PfCSP-Tc/Pb parasites in the vaccinated mice^a^.

Prime	Boost	Number of protected mice (%)			
		Expt. 1	Expt. 2	Expt. 3	Overall
BES-GL3	BES-GL3	0/11 (0)	0/10 (0)	2/7 (29)	2/28 (7)^b^
emBDES	emBDES	0/10 (0)	—	—	0/10 (0)
emBDES	AdHu5	2/10 (20)	—	—	2/10 (20)
AdHu5	emBDES	5/10 (50)^c^	12/15 (80)^c^	9/9 (100)^c^	26/34 (77)^b,c^
AdHu5	AdHu5	2/10 (20)	—	—	2/10 (20)

^a^AdHu5-PfCSP and emBDES-PfCSP/IL12 are showed as AdHu5 and emBDES, respectively. Mice were checked for PfCSP-Tc/Pb blood-stage infections by microscopic examination of Giemsa-stained thin smears of tail blood after challenge infections. Protection is defined as the complete absence of blood-stage parasitaemiae on day 14 post-challenge.

^b^Cumulative data from three independent experiments.

^c^Each group of immunized mice was compared with the control group (BES-GL3) to test for statistically significant differences using Fisher’s exact probability test (*p* < 0.01).

### Immunogenicity of heterologous prime-boost regimens using ChAd63-PfCSP and emBDES-PfCSP/IL12

Although the heterologous AdHu5-PfCSP-prime/emBDES-PfCSP/IL12-boost regimen was highly protective, one of the major problems with AdHu5 is the high seroprevalence of anti-AdHu5 Abs in the human population, particularly in sub-Saharan Africa^36^, where a malaria vaccine is urgently needed. Therefore, we next tested an alternative adenovirus, replication-deficient chimpanzee adenovirus 63 (ChAd63), because ChAd63 has an excellent safety profile with low levels of pre-existing neutralizing Abs against the vector in the African populations^37,38^.

**Figure 5:**
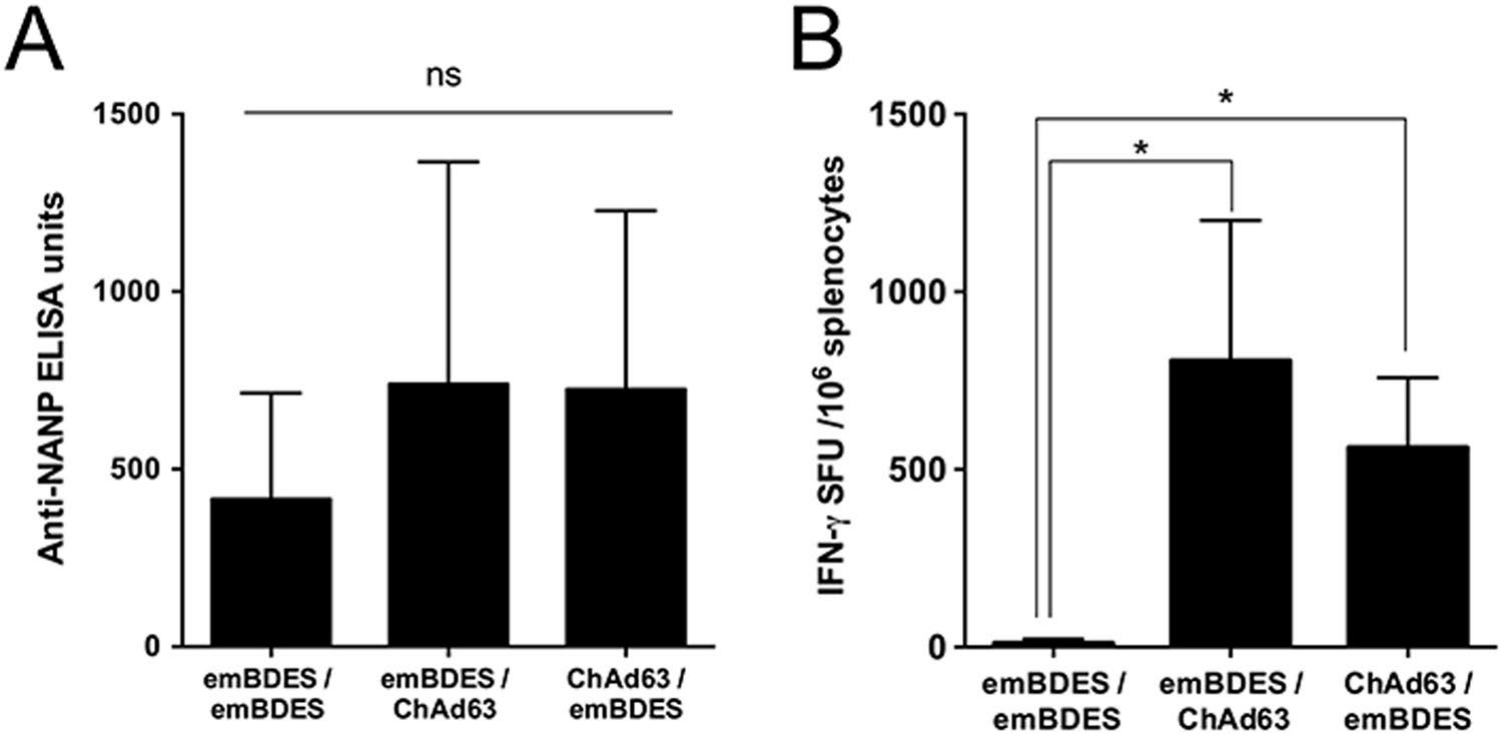
PfCSP-specific Abs and cellular immune responses in mice immunized with heterologous prime-boost regimens using ChAd63-PfCSP and emBDES-PfCSP/IL12. (**A**) Eight BALB/c mice were immunized with the indicated regimens. ChAd63-PfCSP and emBDES-PfCSP/IL12 are shown as ChAd63 and emBDES, respectively. Two weeks after boosting, individual sera were collected and the anti-NANP IgG Ab titres were measured with an ELISA. (**B**) Spleens were collected 2 weeks after boosting, and antigen-specific IFN-γ-secreting T cells were assayed with an *ex vivo* IFN-γ ELISPOT using the PfCSP-overlapping peptide pool. The IFN-γ SFU that reacted with the synthetic peptide per million splenocytes are shown. Bars and error bars indicate the means and SD, respectively. The *p* values were determined with the Mann-Whitney test. *p < 0.05; n = 3 or 4.

**Figure 6:**
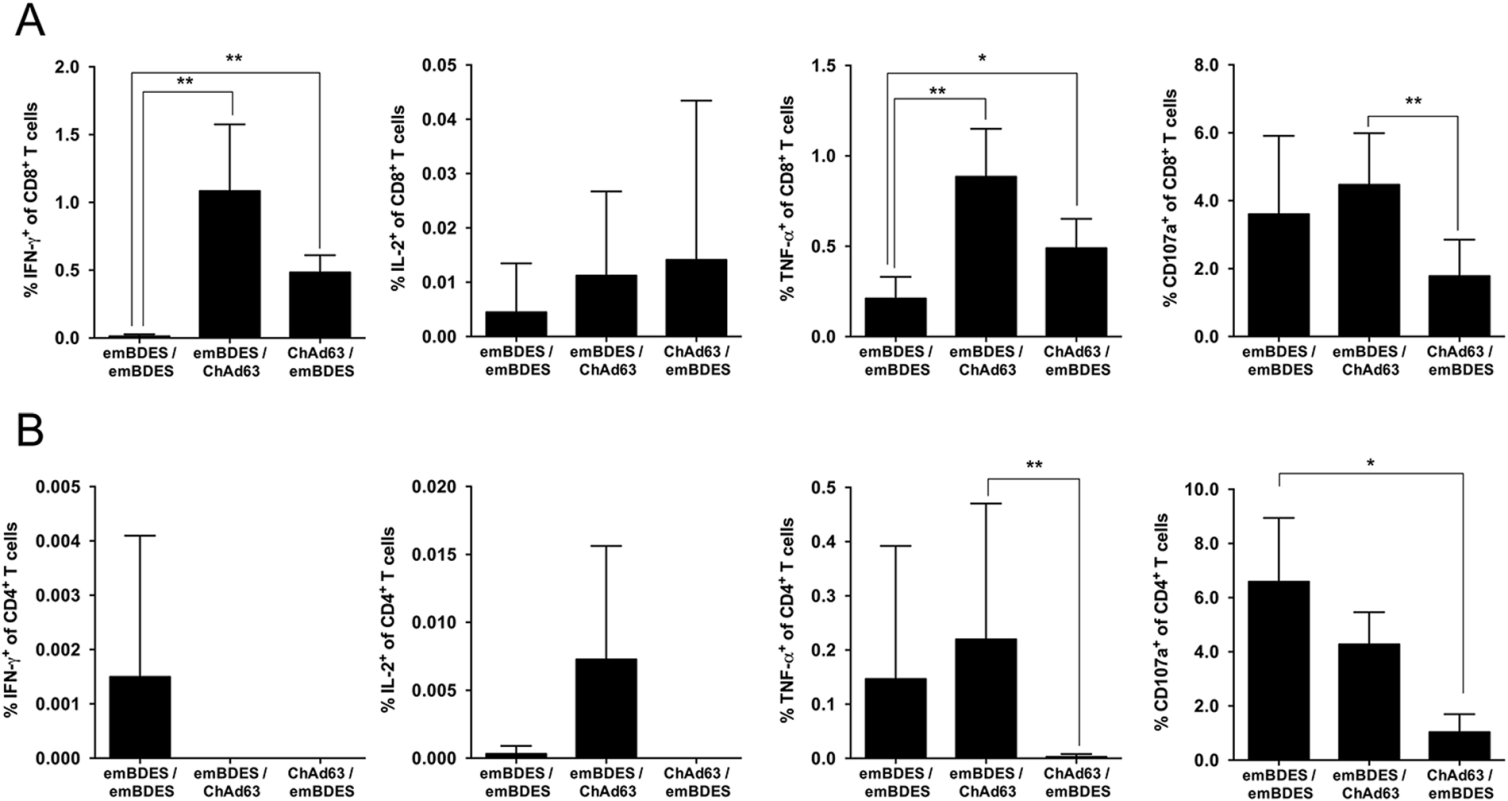
Cellular immune responses in mice immunized with heterologous prime-boost regimens using ChAd63-PfCSP and emBDES-PfCSP/IL12. BALB/c mice were immunized with the indicated regimens. ChAd63-PfCSP and emBDES-PfCSP/IL12 are shown as ChAd63 and emBDES, respectively. Two weeks after boosting, individual blood samples were taken and the frequencies of PfCSP-specific CD8^+^ (**A**) and CD4^+^ (**B**) T cells expressing IFN-γ, TNF-α, IL-2, or CD107a that reacted with the PfCSP-overlapping peptide pool were assayed with ICS. The results are shown as the percentages of CD8^+^ and CD4^+^ T cells secreting each cytokine, and bars and error bars indicate means and SD, respectively. The *p* values were determined with the Mann-Whitney test. *p < 0.05, **p < 0.01; n = 8.

BALB/c mice were immunized intramuscularly (i.m.) with multiple heterologous and homologous regimens using ChAd63-PfCSP and emBDES-PfCSP/IL12 at 3-week intervals. Two weeks after boosting, their sera were collected and examined for Ab titres against the NANP repeat, which is the immunodominant B-cell epitope of PfCSP. Higher Ab titres were induced by ChAd63-PfCSP-prime/emBDES-PfCSP/IL12-boost regimen and by emBDES-PfCSP/IL12-prime/ChAd63-PfCSP-boost regimen than the homologous regimen using emBDES-PfCSP/IL12, although there was no significant differences between these regimens (Fig. 5A). Cellular immune responses in mice were quantified using an *ex vivo* IFN-γ ELISPOT assay (Fig. 5B). The mean IFN-γ SFU per million splenocytes of ChAd63-PfCSP-prime/emBDES-PfCSP/IL12-boost regimen or emBDES-PfCSP/IL12-prime/ChAd63-PfCSP-boost regimen were 806 or 562, respectively (not statistically different). We then did an ICS analysis to quantify the production of various cytokines by a single cell. Our gating strategy for flow cytometry focused on either blood CD8^+^ or CD4^+^ T cells releasing IFN-γ, tumour necrosis factor α (TNF-α), or IL-2, or expressing CD107a, a marker of the degranulation of lymphocytes^39,40^. As shown in the CD8^+^ T-cell panel in Fig. 6A, emBDES-PfCSP/IL12-prime/ChAd63-PfCSP-boost regimen induced higher total IFN-γ, TNF-α, and CD107a (but not IL-2) in response to a pool of overlapping peptides together constituting full-length PfCSP. The total frequency of IFN-γ-secreting cells showed a similar tendency to that identified with ELISPOT. The CD4^+^ T-cell panel in Fig. 6B shows that more cells secreted TNF-α after emBDES-PfCSP/IL12-prime/ChAd63-PfCSP-boost regimen than any other regimen, but there were no significant differences between the cells secreted the other cytokines. A Boolean combination gate analysis was performed with the FlowJo software to sort the cells into either CD8^+^ or CD4^+^ T cells secreting either one, two, or three cytokines (IFN-γ, TNF-α, and/or IL-2) and/or expressing CD107a after peptide stimulation of PBMC from immunized mice (Supplementary Fig. S2). In both cell types, the numbers of single-cytokine-releasing cells were very low and similar. CD8^+^ T cells producing and expressing three markers (IFN-γ, TNF-α, and CD107a) after emBDES-PfCSP/IL12-prime/ChAd63-PfCSP-boost regimen or after ChAd63-PfCSP-prime/emBDES-PfCSP/IL12-boost regimen showed high frequencies, whereas high frequencies of CD4^+^ T cells expressing CD107a and TNF-α were induced by homologous emBDES-PfCSP/IL12 regimen or heterologous emBDES-PfCSP/IL12-prime/ChAd63-PfCSP-boost regimen.

### Protective efficacy of heterologous ChAd63-PfCSP-prime and emBDES-PfCSP/IL12-boost regimens against intravenous (i.v.) sporozoite injection

**Figure 7:**
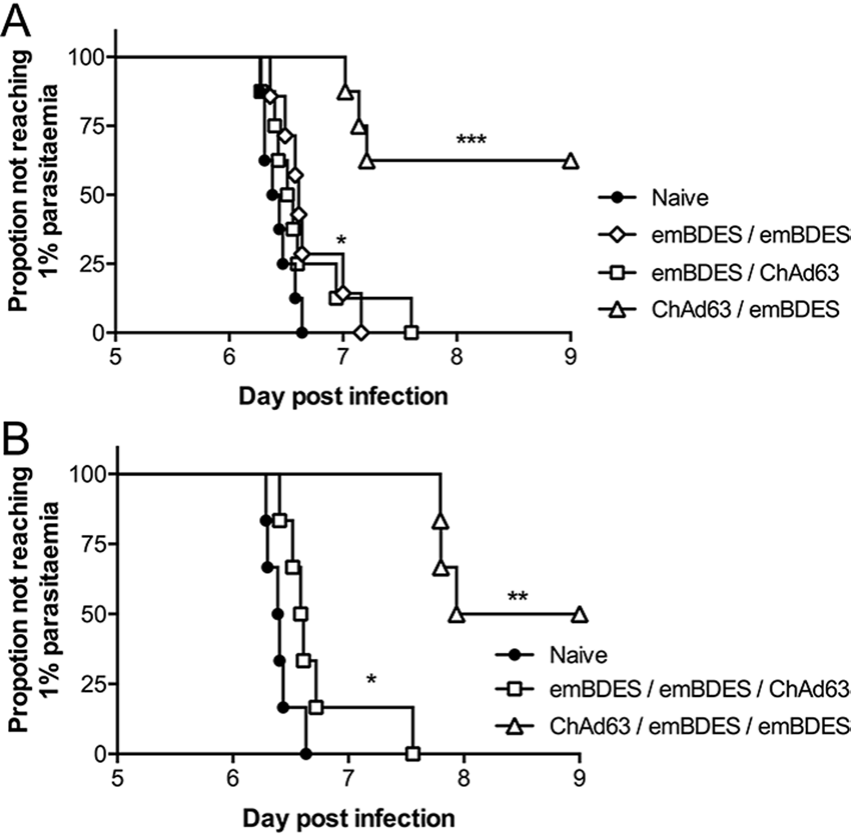
Protective efficacy in mice immunized with heterologous prime-boost regimens using ChAd63-PfCSP and emBDES-PfCSP/IL12. BALB/c mice were immunized with two doses (**A**) (n = 8) or three doses (**B**) (n = 6) of the indicated regimens, and challenged with an intravenous injection of 1,000 transgenic PfCSP@PbCSP (2257cl2) sporozoites. ChAd63-PfCSP and emBDES-PfCSP/IL12 are shown as ChAd63 and emBDES, respectively. Parasitaemiae was monitored for 3 consecutive days, starting from day 5 after challenge, and a model predicting the time to reach 1% parasitaemiae was generated. The absence of blood-stage parasites in the animals that remained sterilely protected on day 8 was confirmed on day 14 after challenge. The statistical analysis was performed with Kaplan-Meier survival curves, and *p* values were calculated with a Kaplan-Meier log-rank (Mantel-Cox) test. ***p < 0.001, **p < 0.01, and *p < 0.05, compared with naïve groups.

The protective efficacy of various heterologous or homologous prime-boost regimens using ChAd63-PfCSP and emBDES-PfCSP/IL12 was assessed by i.v. challenge with 1,000 transgenic PfCSP@PbCSP (2257cl2) sporozoites expressing full-length PfCSP in place of native PbCSP. Consistent with the AdHu5-PfCSP-prime/emBDES-PfCSP/IL12-boost regimen, the ChAd63-PfCSP-prime/emBDES-PfCSP/IL12-boost regimen conferred significantly higher sterile protection (62.5%) than the other combinations (Fig. 7A). The reverse-ordered vaccination (emBDES-PfCSP/IL12-prime/ChAd63-PfCSP-boost regimen) and homologous emBDES-PfCSP/IL12 regimen both showed no protective efficacy. Infected mice immunized with ChAd63-PfCSP-prime/emBDES-PfCSP/IL12-boost regimen showed a significant delay in the time to 1% parasitaemiae, with a mean delay of 0.93 days, compared with the mice immunized with emBDES-PfCSP/IL12-prime/ChAd63-PfCSP-boost regimen (0.24 days) or homologous emBDES-PfCSP/IL12 regimen (0.27 days) (Fig. 7A). We performed additional emBDES-PfCSP/IL12 boosting immunization regimens comprising emBDES-PfCSP/IL12 priming followed by emBDES-PfCSP/IL12 and ChAd63-PfCSP boosting (BBC) or ChAd63-PfCSP priming followed by two doses of emBDES-PfCSP/IL12 boosting (CBB) (Fig. 7B). Compared with the ChAd63-PfCSP-prime/emBDES-PfCSP/IL12-boost regimen, neither BBC (0%) nor CBB (50%) induced further sterile protection, although CBB showed a marked delay in the time to 1% parasitaemiae (mean delay of 1.44 days; *p* = 0.0005).

## Discussion

In this study, we have demonstrated that an adenovirus-prime/emBDES-boost regimen induces hybrid immune responses consisting of humoral and cellular responses to PfCSP, and affords high sterile protection against sporozoite challenge *via* two different routes, infectious mosquito bites (77% protection) and the i.v. injection of sporozoites (62.5% protection), under different conditions at various institutes, including Kanazawa University, Imperial College London, and the Jenner Institute. In many experiments, challenge with i.v. injection has been preferentially used in a range of institutes because the sporozoite dose can be predetermined, even though it is not the natural mode of sporozoite infection and does not involve traversal through the skin. Consequently, the i.v. sporozoite challenge model does not assess the Ab activity induced by vaccines in the skin traversal phase. In contrast, in phase I clinical trials, vaccinated volunteers are challenged by infectious mosquito bites and the salivary glands of all the blood-engorged mosquitoes are dissected to confirm the presence of sporozoites. However, this process is laborious, technically difficult, and time-consuming. Ideally, both routes of sporozoite challenge should be used to evaluate vaccine efficacy in an animal model before a clinical trial. The present study demonstrates the high protective efficacy of adenovirus-prime/emBDES-boost regimen using both challenge models. Thus, our consortium has successfully developed a new vaccine platform based on ChAd63-prime/emBDES-boost regimen, which were originally developed by the Jenner Institute and Kanazawa University, respectively. To our knowledge, this is the first report of a malaria pre-erythrocytic vaccine that is highly effective against sporozoite challenge *via* two different routes.

Heterologous prime-boost vaccination with ChAd63 and MVA expressing the leading pre-erythrocytic antigen, ME-TRAP, is clinically the most potent inducer of CD8^+^ T cells in humans and the most effective malaria vaccine after RTS,S, demonstrating efficacy (defined as sterile protection or delay) in seven of 15 malaria-naive volunteers (46.7%) after controlled human malaria infection^41^. Using the same vaccine platform, ChAd63-MVA expressing CSP reduced the liver parasite burden, but the vaccine efficacy was only 26.7% (4/15)^41^. In BALB/c murine models, the ChAd63-prime/MVA-boost regimen has typically shown only modest protective efficacy (37.5%) against i.v. inoculation with 1,000 sporozoites^42^. Using the same i.v. sporozoite challenge reported in these previous studies, emBDES boost instead of MVA boost in the present study has increased the protective efficacy achieved (62.5%). emBDES has great potential utility in alternative prime-boost combinations with adenoviral-vectored vaccine. Moreover, compared with other viral-vectored vaccines, emBDES has several advantages as a new vaccine platform, especially from the biological safety perspective, including its (i) low cytotoxicity, (ii) inability to replicate in mammalian cells, (iii) absence of pre-existing Abs^43^, (iv) cost-effectiveness and (v) easy manipulation, without an adjuvant formulation^44^.

A fundamental question is why the adenovirus-prime/emBDES-boost heterologous regimen is more effective than the emBDES-prime/adenovirus-boost regimen or homologous prime-boost regimen with the same vaccines. Generally, booster immunizations increase memory CD8^+^ T-cell numbers, with the majority of these cells displaying an effector phenotype and being localized to the peripheral tissues. However, the mechanism that makes heterologous prime-boost more effective than homologous prime-boost is largely unknown. A homologous regimen with AdHu5-PfCSP displayed low protective efficacy despite a very high frequency of antigen-specific T cells (mean IFN-γ-secreting T-cell count after AdHu5-PfCSP, 2,648). Several reports have shown that multifunctional T cells, which can secrete multiple cytokines simultaneously, can contribute to disease because they secrete more IFN-γ on a per cell basis than monofunctional T cells^45,46^. However, there was no correlation between multifunctional CD4^+^ or CD8^+^ T cells and the protection conferred against malaria sporozoites in this study. Therefore, we performed extensive immunological analyses using ELISPOT, ICS, and ELISA with various peptides, but found no reliable surrogate marker that is strongly linked to the protective efficacy induced by adenovirus-prime/emBDES-boost regimen. We only found that the anti-PfCSP IgG2a induced by the AdHu5-PfCSP-prime/emBDES-PfCSP/IL12-boost regimen was higher in the protected mice than in the unprotected mice. Mouse IgG2a is known as a cytophilic Ab, which fixes complement and opsonizes pathogens for phagocytosis more effectively than IgG1^47^. In humans, high levels of IgG Abs to PfCSP have been detected in malaria-endemic populations and these IgG Abs were almost exclusively of the IgG1 and IgG3 subclasses^48^, which have cytophilic activity in cooperation with blood monocytes in the Ab-dependent cellular cytotoxocity effect. Consistent with our data, a previous study showed that mice primed with an anti-tuberculosis DNA vaccine expressing ESAT6 and boosted with the same recombinant protein increased the IgG2a/IgG1 ratio as well as IFN-γ production^49^. It has been reported that the anti-PfCSP IgG isotypes induced by RTS,S in naïve adults were IgG1 and IgG2, and that IgG1 was the dominant subclass, whereas minimal IgG3 and IgG4 responses were detected^50,51^. Therefore, the correlationship between protection and IgG2a suggests that IgG2a is a key factor in the protection induced against skin-stage parasites following the mosquito-bite challenge. Further studies should focus on the mechanisms exploited by the heterologous prime-boost vaccination approach, particularly the innate immune responses induced by subsequent boosting with emBDES.

In summary, we have demonstrated the potential utility of emBDES vectors as an extremely effective booster when combined with adenovirus in a malaria vaccine. The heterologous adenovirus-prime/emBDES-boost regimen shows great potential in inducing unique immune responses, conferring improved immunogenicity and protection against sporozoites delivered by both mosquito-bite and i.v. injection. This study is the first step toward the development of a new malaria vaccine platform based on adenovirus-prime/emBDES-boost immunization regimen. It will also be very interesting to apply this vaccine platform to other infectious diseases that have an intracellular stage, as well as malaria parasites.

## Materials and Methods

### Animals, cell lines, peptides, and parasites

Female inbred BALB/c (*H-2*^*d*^) mice were used to assess immunogenicity and protection. *Spodoptera frugiperda* (Sf9) and HEK293A cells were maintained as described previously^10^. Recombinant PfCSP (rPfCSP) was produced in *Escherichia coli*, as described previously^10^. The synthetic peptides (NANP_6_C repeat, NYDNAGTNL, and 20-mer peptide pools overlapping PfCSP by 10 amino acids) have been described previously^10,52^. Transgenic *P. berghe*i lines expressing the full-length *PfCSP* gene of strain 3D7, designated PfCSP-Tc/Pb and PfCSP@PbCSP (2257cl2) [(PbANKA-PfCSP(r)PbCSP GFP::LucPbeef1a; line RMgm-4110; www.pberghei.eu) have been described previously^53,54^.

### Ethics statement

All animal care and handling procedures were approved by the Animal Care and Ethical Review Committee of Kanazawa University (no. 22118–1). All procedures were performed in accordance with the UK Animals (Scientific Procedures) Act (PPL 70/8788, Imperial College; PPL 30/2414, Oxford). All activities at Imperial College were approved by the Imperial College’s Animal Welfare and Ethical Review Body.

### Recombinant viral vaccines

BDES-sPfCSP2-WPRE-Spider, BES-mIL-12-Spider, and BES-GL3 have been described previously^16,17^. In this paper, the BDES-sPfCSP2-WPRE-Spider and BDES vaccines consisting of BDES-sPfCSP2-WPRE-Spider and BES-mIL-12-Spider are described as emBDES-PfCSP and emBDES-PfCSP/IL12, respectively. To generate the AdHu5-PfCSP, the gene cassette encoding GPI-anchor lacking PfCSP (Leu19-Val377) fused to the VSV-G protein membrane anchor sequence, followed by a *wpre* sequence, was excised from pFast-sPfCSP2-WPRE-Spider^16^ by digestion with *Eco*RI and *Xho*I and then inserted into the *EcoR*I and *Xho*I sites of pAd/PL-DEST (Invitrogen, Carlsbad, CA, USA) under the control of the CAG promoter sequence. The adenovirus was purified and titrated with the Fast-Trap Adenovirus Purification and Concentration Kit (Millipore, Temecula, CA, USA) and the Adeno-X™ Rapid Titer Kit (Clontech, Palo Alto, CA, USA), respectively, according to the manufacturers’ protocols. ChAd63-PfCSP encoding PfCSP (Leu19-Ser383) sequence has been described previously^55^. All *PfCSP* genes inserted into BDES-sPfCSP2-WPRE-Spider, AdHu5-PfCSP, or ChAd63-PfCSP were codon optimized.

### *In vivo* bioluminescent imaging

Luciferase-expressing BES-GL3 was administered into the right tibialis anterior muscle of BALB/c mice (n = 3; 1 × 10^8^ pfu/mouse) on day 0, and at the appropriate time points, d-luciferin (15 mg/ml; OZ Biosciences, Marseille, France) was administered intraperitoneally (150 μl/mouse). After 10 min, the animals were anesthetized with a ketamine (100 mg/kg)/xylazine (10 mg/kg) mixture, and the luciferase expression was detected with an IVIS^®^ Lumina LT *in vivo* imaging system (PerkinElmer, Waltham, MA, USA). The luciferase expression decreased to an undetectable level by day 42, and BES-GL3 was re-administered into the left tibialis anterior muscle 14 days later (day 56). Luciferase expression was detected as described above. To quantify the luminescence, the region on the captured images was selected, and the luminescence output was analysed with the Living Image software and expressed as the total flux (photons/second). The relative total flux was calculated after the maximum total flux at 3 h defined as 100% and the background total flux was defined as 0%.

### Immunoblotting and IFA assay

All immunoblotting and IFA-related methods used are described in the Supplementary Information.

### ELISA

Sera from the immunized mice were collected as tail blood samples 2–3 weeks after the first and second immunizations and one day before challenge. PfCSP-specific Ab levels were quantified with an ELISA. EIA/RIA plates (Corning Inc.; Corning, NY, USA) pre-coated with GPI-anchor-truncated recombinant PfCSP (rPfCSP; 0.4 µg/well)^10^ or the synthesized repeated-NANP_6_C-sequence peptide (0.1 µg/well) were blocked with 1% bovine serum albumin in phosphate-buffered saline (PBS) and incubated with serial dilutions of sera from the immunized and control mice. To determine the anti-PfCSP isotype IgG Ab titres, the total IgG, IgG1, IgG2a, and IgG2b titres were measured with horseradish peroxidase (HRP)-conjugated goat anti-mouse IgG antibodies. The endpoint titres were expressed as the reciprocal of the last dilution that gave an optical density at 414 nm of 0.15 U above the value of the negative control (<0.1). To determine the anti-PfCSP NANP IgG Ab titres, the total IgG titre was measured with an alkaline-phosphatase-conjugated goat anti-mouse whole-IgG antibody. The results were expressed in ELISA units (EU). A 1:14,000 dilution (63.6 ng/ml) of mouse anti-PfCSP NANP mAb 2A10 had an EU of exactly 1. All the mice used were seronegative before immunization. To determine the anti-baculovirus IgG Ab titres, the total IgG titres were measured with an HRP-conjugated goat anti-mouse IgG antibody. The endpoint titres were expressed as the reciprocal of the last dilution that gave an optical density at 414 nm of 0.15 U above the value of the negative control (<0.1).

### *Ex vivo* IFN-γ ELISPOT assay

Ammonium chloride potassium lysing buffer (ACK)-treated splenocytes were cultured for 20–24 h on MAIP ELISPOT plates (Mabtech, Stockholm, Sweden) or BD ELISPOT plates (BD Bioscience, San Jose, CA) with the immunodominant *H-2K*^*d*^-restricted T-cell epitope (NYDNAGTNL, PfCSP_39-47_; final concentration, 1 µg/ml) or the PfCSP-overlapping peptide pool (final concentration, 5 µg/ml). The IFN-γ ELISPOT assay was conducted with coating and detecting mAbs from the IFN-γ ELISPOT ALP Kit (Mabtech) or the BD™ IFN-γ ELISPOT Set (BD Biosciences), according to the manufacturer’s protocol. Spots were counted with an ELISPOT plate counter (Autoimmun Diagnostika, Strassberg, Germany) and expressed as IFN-γ SFU per million splenocytes, after the background subtraction of wells containing cells and medium, but no peptide.

### Intracellular cytokine staining (ICS)

ICS was performed with splenocytes and peripheral blood mononuclear cells (PBMCs), as described previously^30^. The splenocytes were stimulated with a final concentration of 1 µg/ml of the immunodominant CD8^+^ T-cell epitope NYDNAGTNL (PfCSP_39-47_) and 1 µg/ml of GolgiPlug™ (BD Biosciences) in a 96-well U-bottom tissue culture plate (Corning Inc.) for 6 h. The cells were then surface stained with anti-mouse CD16/32 Ab, Pacific Blue™-conjugated anti-mouse CD4 Ab, and PerCP/Cy5.5-conjugated anti-mouse CD8α Ab, and the cytokine was stained with fluorescein isothiocyanate (FITC)-conjugated anti-mouse IFN-γ Ab or a FITC-conjugated rat IgG1κ Isotype control Ab. Data were acquired with a BD FACSVerse™ Flow Cytometer (BD Biosciences) and analysed with FlowJo (Tree Star, Ashland, OR, USA). All Abs were purchased from BioLegend (San Diego, CA, USA). The PMBCs were stimulated with a final concentration of 5 µg/ml of the PfCSP-overlapping peptide, 1 µg/ml of GolgiPlug™ (BD Biosciences), and phycoerythrin-conjugated anti-mouse CD107a Ab in a 96-well U-bottom tissue culture plate (Corning Inc.) for 6 h. The cells were then surface stained with anti-mouse CD16/32 Ab, eFluor-450-conjugated anti-mouse CD4 Ab, and PerCP-cyanine5.5-conjugated anti-mouse CD8α Ab, and the cytokines were stained with allophycocyanin-conjugated anti-mouse IFN-γ Ab, Alexa-Fluor-488-conjugated anti-mouse TNFα Ab, and PE-cyanine7-conjugated anti-mouse IL-2 Ab. The data were acquired with a BD LSR II Flow Cytometer (BD Biosciences) and analysed with the FlowJo software. All Abs were purchased from eBioscience.

### Immunization and challenge infections

#### Protective efficacy of heterologous AdHu5-PfCSP-prime/emBDES-PfCSP/IL12-boost immunization against mosquito-bite challenge

Balb/c mice were immunized i.m. twice, with a 3-week interval between the primary and booster immunizations. The doses of vaccines were 100 µl containing 5 × 10^7^ plaque-forming units (pfu) for AdHu5-PfCSP and 100 µl containing 2 × 10^8^ pfu for emBDES-PfCSP/IL12. Two weeks after the booster immunization, the mice were challenged with bites of PfCSP-Tc/Pb-infected mosquitoes, as described previously^10^. Briefly, infected mosquitoes were allowed to feed on the abdomen of each mouse for 15 min. To ensure a high probability of baseline infection (in the control mice), all the mice received a minimum of two bites and a maximum of seven bites. The salivary glands from all the blood-engorged mosquitoes in the control group were dissected to confirm the presence of sporozoites, indicating a potentially infective bite. Where necessary, mosquito biting was performed repeatedly until 2–7 infected mosquitoes had bitten each mouse. The mice were checked for *P. berghei* blood-stage infection with a microscopic examination of Giemsa-stained thin smears of their tail blood, prepared on days 5, 7, 9 11, and 14 after challenge. A minimum of 20 fields (magnification, ×1,000) were examined before a mouse was deemed to be negative for infection. Protection was defined as the complete absence of blood-stage parasitaemiae on day 14 after challenge.

#### Protective efficacy of heterologous ChAd63-prime/emBDES-boost immunization against intravenous sporozoite challenge

Balb/c mice were immunized i.m. twice, with a 3-week interval between the primary and booster immunizations. The doses of vaccines were 50 µl containing 1 × 10^8^ pfu for ChAd63-PfCSP and 100 µl containing 2 × 10^8^ pfu for emBDES-PfCSP/IL12. Two weeks after the booster immunizations, the mice were challenged with 1,000 PfCSP@PbCSP (2257cl2) sporozoites *via* the i.v. route into the tail vein, as described previously^30^. In a parallel experiment, Balb/c mice were immunized three times at 3-week intervals *via* the i.m. route, and were challenged with sporozoites *via* the i.v. route 8 days after the final immunization. The mice were checked for *P. berghei* blood-stage infection by the microscopic examination of Giemsa-stained thin smears of their tail blood, prepared on days 5, 6, 7, 8, 11, and 14 after challenge. The time required to reach 1% parasitaemiae was determined as described previously^34^. A minimum of 20 fields (magnification, ×1,000) were examined before a mouse was deemed to be negative for infection. Protection was defined as the complete absence of blood-stage parasitaemiae on day 14 after challenge.

### Statistical analysis

A two-tailed Fisher’s exact probability test was performed to determine the significance of differences in the protective efficacies of the vaccines, using the SPSS software (version 19, Chicago, IL, USA). In all other experiments, statistical differences between the experimental groups were analysed by the methods described in the individual figure legends; *p* values of <0.05 were considered statistically significant. Statistical analyses were performed with either Prism version 7.0a (GraphPad Software Inc., La Jolla, CA, USA) or Microsoft® Excel (Radmond, WA, USA).

## Electronic supplementary material

Supplementary Information

## References

[1] WHO. World malaria report 2017. *World Health Organization, Geneva, Switzerland*, http://www.who.int/malaria/publications/world-malaria-report-2017/en/ (2017).

[2] Minsoko PA (2014). Efficacy and Safety of the RTS,S/AS01 Malaria Vaccine during 18 Months after Vaccination: A Phase 3 Randomized, Controlled Trial in Children and Young Infants at 11 African Sites. PloS Med.

[3] Tinto H (2015). Efficacy and safety of RTS,S/AS01 malaria vaccine with or without a booster dose in infants and children in Africa: final results of a phase 3, individually randomised, controlled trial. Lancet.

[4] White MT (2015). Immunogenicity of the RTS,S/AS01 malaria vaccine and implications for duration of vaccine efficacy: secondary analysis of data from a phase 3 randomised controlled trial. Lancet Infect Dis.

[5] White MT (2013). The relationship between RTS,S vaccine-induced antibodies, CD4(+) T cell responses and protection against Plasmodium falciparum infection. PLoS One.

[6] Ockenhouse CF (2015). Ad35.CS.01 - RTS,S/AS01 heterologous prime boost vaccine efficacy against sporozoite challenge in healthy malaria-naive adults. PLoS One.

[7] Kazmin D (2017). Systems analysis of protective immune responses to RTS,S malaria vaccination in humans. Proc Natl Acad Sci USA.

[8] Yoshida S, Kawasaki M, Hariguchi N, Hirota K, Matsumoto M (2009). A baculovirus dual expression system-based malaria vaccine induces strong protection against *Plasmodium berghei* sporozoite challenge in mice. Infect Immun.

[9] Mizutani M (2014). Baculovirus-vectored multistage *Plasmodium vivax* vaccine induces both protective and transmission-blocking immunities against transgenic rodent malaria parasites. Infect Immun.

[10] Iyori M (2013). Protective efficacy of baculovirus dual expression system vaccine expressing *Plasmodium falciparum* circumsporozoite protein. PLoS One.

[11] Yoshida S (2010). *Plasmodium berghei* circumvents immune responses induced by merozoite surface protein 1- and apical membrane antigen 1-based vaccines. PLoS One.

[12] Yoshida S, Araki H, Yokomine T (2010). Baculovirus-based nasal drop vaccine confers complete protection against malaria by natural boosting of vaccine-induced antibodies in mice. Infect Immun.

[13] Sala KA (2015). The *Plasmodium berghei* sexual stage antigen PSOP12 induces anti-malarial transmission blocking immunity both *in vivo* andi*n vitro*. Vaccine.

[14] Blagborough AM, Yoshida S, Sattabongkot J, Tsuboi T, Sinden RE (2010). Intranasal and intramuscular immunization with Baculovirus Dual Expression System-based Pvs25 vaccine substantially blocks *Plasmodium vivax* transmission. Vaccine.

[15] Mlambo G, Kumar N, Yoshida S (2010). Functional immunogenicity of baculovirus expressing Pfs25, a human malaria transmission-blocking vaccine candidate antigen. Vaccine.

[16] Iyori M (2017). DAF-shielded baculovirus-vectored vaccine enhances protection against malaria sporozoite challenge in mice. Malar J.

[17] Iyori, M. *et al*. Protective efficacy of an IL-12-expressing baculoviral malaria vaccine. *Parasite Immunol* **39** (2017). 10.1111/pim.12498 29072334

[18] McShane H, Brookes R, Gilbert SC, Hill AV (2001). Enhanced immunogenicity of CD4( + ) t-cell responses and protective efficacy of a DNA-modified vaccinia virus Ankara prime-boost vaccination regimen for murine tuberculosis. Infect Immun.

[19] Brooks JV, Frank AA, Keen MA, Bellisle JT, Orme IM (2001). Boosting vaccine for tuberculosis. Infect Immun.

[20] Santosuosso M, McCormick S, Zhang X, Zganiacz A, Xing Z (2006). Intranasal boosting with an adenovirus-vectored vaccine markedly enhances protection by parenteral Mycobacterium bovis BCG immunization against pulmonary tuberculosis. Infect Immun.

[21] Dean G (2014). Comparison of the immunogenicity and protection against bovine tuberculosis following immunization by BCG-priming and boosting with adenovirus or protein based vaccines. Vaccine.

[22] Xing Z (2009). Intranasal mucosal boosting with an adenovirus-vectored vaccine markedly enhances the protection of BCG-primed guinea pigs against pulmonary tuberculosis. PLoS One.

[23] Roediger EK, Kugathasan K, Zhang X, Lichty BD, Xing Z (2008). Heterologous boosting of recombinant adenoviral prime immunization with a novel vesicular stomatitis virus-vectored tuberculosis vaccine. Mol Ther.

[24] Takeda A (2003). Protective efficacy of an AIDS vaccine, a single DNA priming followed by a single booster with a recombinant replication-defective Sendai virus vector, in a macaque AIDS model. J Virol.

[25] Matoba N (2006). Humoral immune responses by prime-boost heterologous route immunizations with CTB-MPR(649-684), a mucosal subunit HIV/AIDS vaccine candidate. Vaccine.

[26] Sullivan NJ (2003). Accelerated vaccination for Ebola virus haemorrhagic fever in non-human primates. Nature.

[27] Warfield KL (2015). Homologous and heterologous protection of nonhuman primates by Ebola and Sudan virus-like particles. PLoS One.

[28] Reyes-Sandoval A (2010). Prime-boost immunization with adenoviral and modified vaccinia virus Ankara vectors enhances the durability and polyfunctionality of protective malaria CD8^+^ T-cell responses. Infect Immun.

[29] Capone S (2010). Immune responses against a liver-stage malaria antigen induced by simian adenoviral vector AdCh63 and MVA prime-boost immunisation in non-human primates. Vaccine.

[30] Bauza K (2014). Efficacy of a *Plasmodium vivax* malaria vaccine using ChAd63 and modified vaccinia Ankara expressing thrombospondin-related anonymous protein as assessed with transgenic *Plasmodium berghei* parasites. Infect Immun.

[31] Van Braeckel-Budimir N, Kurup SP, Harty JT (2016). Regulatory issues in immunity to liver and blood-stage malaria. Curr Opin Immunol.

[32] Reyes-Sandoval A (2011). CD8^+^ T effector memory cells protect against liver-stage malaria. J Immunol.

[33] Overstreet MG, Cockburn IA, Chen YC, Zavala F (2008). Protective CD8 T cells against *Plasmodium* liver stages: immunobiology of an ‘unnatural’ immune response. Immunol Rev.

[34] Epstein JE (2011). Live attenuated malaria vaccine designed to protect through hepatic CD8 T cell immunity. Science.

[35] Schmidt NW, Butler NS, Badovinac VP, Harty JT (2010). Extreme CD8 T cell requirements for anti-malarial liver-stage immunity following immunization with radiation attenuated sporozoites. PLoS Pathog.

[36] Nwanegbo E (2004). Prevalence of neutralizing antibodies to adenoviral serotypes 5 and 35 in the adult populations of The Gambia, South Africa, and the United States. Clin Diagn Lab Immunol.

[37] Dicks MD (2012). A novel chimpanzee adenovirus vector with low human seroprevalence: improved systems for vector derivation and comparative immunogenicity. PLoS One.

[38] Nebie I (2014). Assessment of chimpanzee adenovirus serotype 63 neutralizing antibodies prior to evaluation of a candidate malaria vaccine regimen based on viral vectors. Clin Vaccine Immunol.

[39] Betts MR (2003). Sensitive and viable identification of antigen-specific CD8^+^ T cells by a flow cytometric assay for degranulation. J Immunol Methods.

[40] Alter G, Malenfant JM, Altfeld M (2004). CD107a as a functional marker for the identification of natural killer cell activity. J Immunol Methods.

[41] Hodgson SH (2015). Evaluation of the efficacy of ChAd63-MVA vectored vaccines expressing circumsporozoite protein and ME-TRAP against controlled human malaria infection in malaria-naive individuals. J Infect Dis.

[42] Longley RJ (2015). Comparative assessment of vaccine vectors encoding ten malaria antigens identifies two protective liver-stage candidates. Scientific reports.

[43] Luo WY (2013). Adaptive immune responses elicited by baculovirus and impacts on subsequent transgene expression *in vivo*. J Virol.

[44] Gerster P (2013). Purification of infective baculoviruses by monoliths. Journal of chromatography. A.

[45] Darrah PA (2007). Multifunctional TH1 cells define a correlate of vaccine-mediated protection against *Leishmania major*. Nat Med.

[46] Seder RA, Darrah PA, Roederer M (2008). T-cell quality in memory and protection: implications for vaccine design. Nat Rev Immunol.

[47] Azeredo da Silveira S (2002). Complement activation selectively potentiates the pathogenicity of the IgG2b and IgG3 isotypes of a high affinity anti-erythrocyte autoantibody. J Exp Med.

[48] John CC (2005). Correlation of high levels of antibodies to multiple pre-erythrocytic *Plasmodium falciparum* antigens and protection from infection. Am J Trop Med Hyg.

[49] Wang QM (2004). Improved immunogenicity of a tuberculosis DNA vaccine encoding ESAT6 by DNA priming and protein boosting. Vaccine.

[50] Stoute JA (1997). A preliminary evaluation of a recombinant circumsporozoite protein vaccine against *Plasmodium falciparum* malaria. RTS,S Malaria Vaccine Evaluation Group. N Engl J Med.

[51] Kester KE (2001). Efficacy of recombinant circumsporozoite protein vaccine regimens against experimental *Plasmodium falciparum* malaria. J Infect Dis.

[52] Collins KA, Snaith R, Cottingham MG, Gilbert SC, Hill AVS (2017). Enhancing protective immunity to malaria with a highly immunogenic virus-like particle vaccine. Scientific reports.

[53] Sumitani M (2013). Reduction of malaria transmission by transgenic mosquitoes expressing an antisporozoite antibody in their salivary glands. Insect Mol Biol.

[54] Triller, G. *et al*. Natural parasite exposure induces protective human anti-malarial antibodies. *Immunity* **47**, 1197–1209, e1110 (2017). 10.1016/j.immuni.2017.11.007 PMC5738265 29195810

[55] de Barra E (2014). A phase Ia study to assess the safety and immunogenicity of new malaria vaccine candidates ChAd63 CS administered alone and with MVA CS. PLoS One.

