# Supplementary material for: Adenovirus-prime and baculovirus-boost heterologous immunization achieves sterile protection against malaria sporozoite challenge in a murine model
Source: Sci Rep. 2018 Mar 1;8:3896. doi: 10.1038/s41598-018-21369-y (PMC5832798; doi:10.1038/s41598-018-21369-y)
Supplement: Supplementary file 1 — Supplementary Information [file 41598_2018_21369_MOESM1_ESM.pdf]

Adenovirus-prime and baculovirus-boost heterologous immunization achieves sterile protection against malaria sporozoite challenge in a murine model

Kunitaka Yoshida[1,2], Mitsuhiro Iyori[1], Andrew M. Blagborough[3], Ahmed M. Salman[4,5], Pawan Dulal[4], Katarzyna A. Sala[3], Daisuke S. Yamamoto[6], Shahid M. Khan[5], Chris J. Janse[5], Sumi Biswas[4], Tatsuya Yoshii[1], Yenni Yusuf[1], Masaharu Tokoro[2], Adrian V. S. Hill[4], Shigeto Yoshida[1]*

Supplementary Information

Immunoblotting and IFA assay

For immunoblotting, HEK293A cells (6×10^4) were infected with AdHu5-PfCSP (MOI = 1) or transduced with emBDES-PfCSP (MOI = 100). After 48h incubation, the infected or transduced cells were prepared in reducing Laemmli lysis buffer and boiled for 5 min. The proteins were separated by 10% SDS-PAGE, transferred to an Immobilon-FL PVDF membrane (Merck Millipore, Guyancourt, France), and then probed with an anti-PfCSP monoclonal Ab (mAb) 2A10 (MR4, Manassas, VA). After incubation with IRDye-800-conjugated anti-mouse IgG Ab (Rockland Immunochemicals, Gilbertsville, PA, USA), the bound Abs were detected by electrochemiluminescence (GE Healthcare, Waukesha, WI), as described previously[1]. For IFA, HEK293A cells (3×10^4) were infected with AdHu5-PfCSP (MOI = 1) or transduced with emBDES-PfCSP (MOI = 100). After 48h incubation, the infected or transduced cells were fixed with 5% paraformaldehyde and after blocking with 10% normal goat serum, were incubated with anti-PfCSP mAb 2A10 conjugated with Alexa Fluor 594 for 1 h, washed five times in PBS, and mounted with Vectashield containing 4′,6-diamidino-2-phenylindole (DAPI; Vector Laboratories, Burlingame, CA, USA). A BZ-X700 fluorescence microscope (Keyence, Tokyo, Japan) with a 100× objectives was used for image acquisition.

1 Mizutani, M. *et al.* Development of a *Plasmodium berghei* transgenic parasite expressing the full-length *Plasmodium vivax* circumsporozoite VK247 protein for testing vaccine efficacy in a murine model. *Malar J* **15**, 251 (2016).

Supplementary Figures and Table:

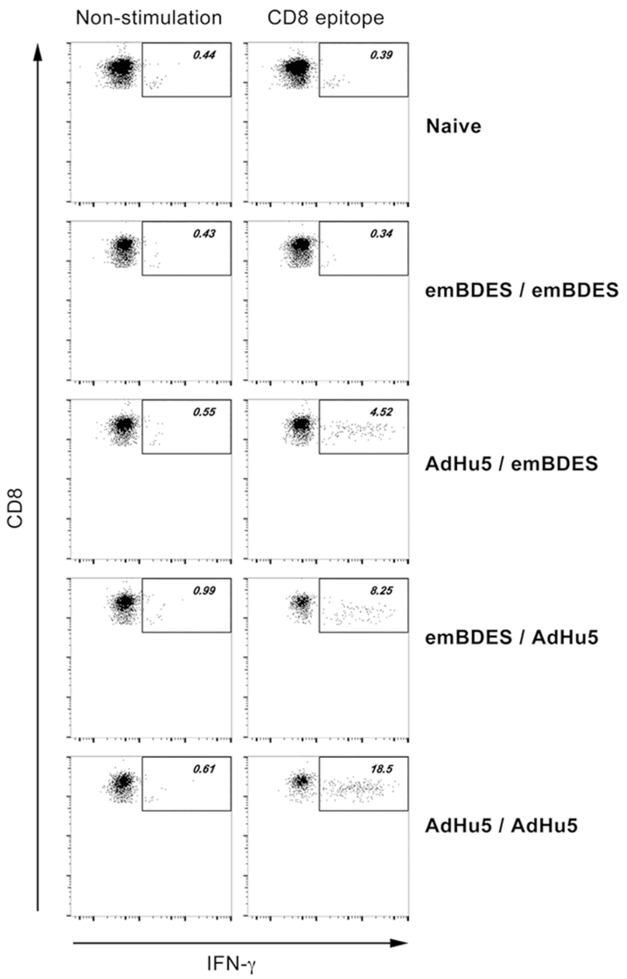

Figure S1. PfCSP-specific cellular immune responses after heterologous prime–boost immunization regimens using AdHu5-PfCSP and emBDES-PfCSP/IL12. BALB/c mice were immunized with the indicated regimens. AdHu5-PfCSP and emBDES-PfCSP/IL12 are shown as AdHu5 and emBDES, respectively. Two weeks after boosting, splenocytes were stimulated with the synthetic PfCSP-specific CD8 epitope. Percentages of IFN-γ-secreting cells in the $CD8^{+}CD4^{-}$ T-cell population were determined after the subtraction of the percentages of cells that stained with an isotype control Ab.

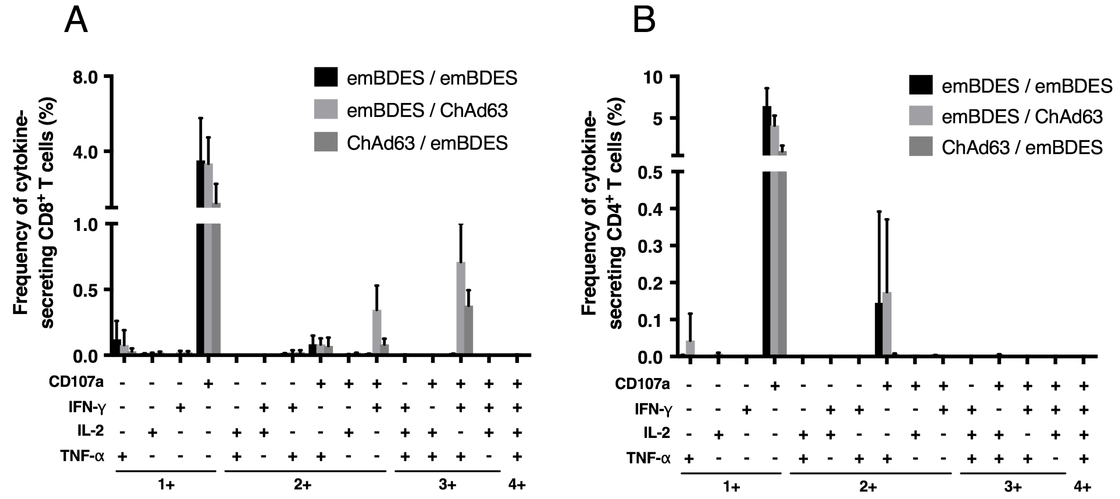

Figure S2. T-cell multifunctionality after heterologous prime–boost immunization regimens using ChAd63-PfCSP and emBDES-PfCSP/IL12. BALB/c mice were immunized with the indicated regimens. ChAd63-PfCSP and emBDES-PfCSP/IL12 are shown as ChAd63 and emBDES, respectively. Single cells from the mouse spleens were stimulated with the peptide pool of PfCSP and stained as described in Fig. 6 and the *Materials and Methods*. Graphs represent the percentages of $CD8^+$ T cells (A) or $CD4^+$ T cells (B) secreting either one, two, or all three cytokines and/or expressing CD107a. Data were analysed with FlowJo.

Supplementary Table S1. The mean numbers of infectious bites per mouse in Table 1.

Prime	Boost	Mean numbers of infectious bites ± S.D. (min number – max number)		
		**Expt 1[a]**	**Expt 2[b]**	**Expt 3[b]**
BES-GL3	BES-GL3	2.9 ± 1.0 (2 - 5)	3.6 ± 0.8 (3 - 5)	3.3 ± 0.8 (2 - 4)
emBDES	emBDES	3.1 ± 1.2 (2 - 5)	-	-
emBDES	AdHu5	3.8 ± 1.1 (2 - 6)	-	-
AdHu5	emBDES	2.9 ± 0.6 (2 - 4)	3.9 ± 1.2 (3 - 7)	2.7 ± 0.7 (2 - 4)
AdHu5	AdHu5	3.5 ± 1.4 (2 - 6)	-	-

[a] No significant difference among the groups by a Kruskal-Wallis test.

[b] No significant difference between the groups by a Mann-Whitney test.
